# Effects of early social deprivation on epigenetic statuses and adaptive behavior of young children: A study based on a cohort of institutionalized infants and toddlers

**DOI:** 10.1371/journal.pone.0214285

**Published:** 2019-03-26

**Authors:** Oxana Yu. Naumova, Sergey Yu. Rychkov, Sergey A. Kornilov, Veronika V. Odintsova, Varvara О. Anikina, Maria Yu. Solodunova, Irina A. Arintcina, Marina A. Zhukova, Irina V. Ovchinnikova, Olga V. Burenkova, Olga V. Zhukova, Rifkat J. Muhamedrahimov, Elena L. Grigorenko

**Affiliations:** 1 Human Genetics Laboratory, Vavilov Institute of General Genetics RAS, Moscow, Russian Federation; 2 Department of Psychology, Saint-Petersburg State University, Saint Petersburg, Russian Federation; 3 Department of Psychology, University of Houston, Houston, Texas, United States of America; 4 Department of Biological Psychology, VU University, Amsterdam, Netherlands; 5 National Medical Research Center for Obstetrics, Gynecology and Perinatology, Moscow, Russian Federation; 6 Federal Research Institute for Health Organization and Informatics, Moscow, Russia; 7 Baylor College of Medicine, Houston, Texas, United States of America; University of Bonn, Institute of Experimental Hematology and Transfusion Medicine, GERMANY

## Abstract

Early social deprivation (i.e., an insufficiency or lack of parental care) has been identified as a significant adverse early experience that may affect multiple facets of child development and cause long-term outcomes in physical and mental health, cognition and behavior. Current research provides growing evidence that epigenetic reprogramming may be a mechanism modulating these effects of early adversities. This work aimed to investigate the impact of early institutionalization—the immersion in an extreme socially depriving environment in humans—on the epigenome and adaptive behavior of young children up to 4 years of age. We conducted a cross-sectional study involving two comparison groups: 29 children raised in orphanages and 29 children raised in biological families. Genome-wide DNA methylation profiles of blood cells were obtained using the Illumina MethylationEPIC array; the level of child adaptive functioning was assessed using the Vineland Adaptive Behavior Scales-II. In comparison to children raised in families, children residing in orphanages had both statistically significant deficits in multiple adaptive behavior domains and statistically significant differences in DNA methylation states. Moreover, some of these methylation states may directly modulate the behavioral deficits; according to preliminary estimates, about 7–14% of the deviation of adaptive behavior between groups of children may be determined by their difference in DNA methylation profiles. The duration of institutionalization had a significant impact on both the adaptive level and DNA methylation status of institutionalized children.

## Introduction

Early psychosocial deprivation is a term that is frequently used to describe early developmental environments characterized by insufficient or absent parental care. Early deprivation has been identified as an important risk factor for negative developmental outcomes in a variety of functional domains, from physical and mental health to cognition and behavior. According to the UNICEF Progress for Children report [1], in 2009 nearly 140 million children around the world could be classified as orphans; of those, 15 million had lost both parents. Although most of these children live with other relatives or reside in foster families, a substantial 8 million abandoned or orphaned children were in institutional care (i.e., living in a residential institution with no or limited individualized environment and care) at the beginning of 2009; the situation is unlikely to have changed now. Institutional care is frequently characterized by profound levels of psychosocial deprivation: that is, even when a child's basic physical and educational needs are addressed, institutional environments prohibit the development of early attachment and other crucial relationships between the child and a stable adult caregiver. Such relationships are critical for children's socio-emotional development and well-being, and form the foundational layer for the development of self-regulation and adaptive skills [2].

A substantial body of evidence established in the past two decades has highlighted the negative effects of early institutionalization on a multitude of developmental domains. Children residing in institutions have been reported to show delays in physical growth, deficits in motor development, and profound delays in cognitive functioning and language development [3–7]. These delays and deficits may persist for a long time after a child has been placed into foster care or adopted. Thus, early experience of institutionalization and its duration have been shown to be associated with various developmental disorders and functional deficits in children and adolescents, such as deficits in visual memory and executive functioning [8], attachment disorder behavior [9, 10], internalizing, externalizing, and attention problems [11–13], among others.

The effects of early institutionalization are typically viewed as a combination of the outcomes of early stress, similar to other early adversities, that involves neurobiological mechanisms linking exposure to adverse experiences in childhood to cognitive and behavioral development [14–18]. Rapidly growing evidence provides further support to the hypothesis that genome activity regulation might be one of the main molecular mechanisms underlying stress-induced neurobiological changes, which precedes and modulates phenotypic manifestations [15, 17, 19]. Research has established that variation in social environments might significantly affect the functional activity of the genome through the up- or downregulation of specific gene networks as well as by causing a generalized genomic response. These changes can be traced to epigenetic marks, such as histone acetylation and DNA methylation, which modulate the environmental influences on gene expression and negative developmental outcomes. Two main hypotheses have been put forward to provide a mechanistic explanation of the modulating effects of early adversity on the epigenome. The first hypothesis states that early life adversity leads to the concurrent and independent epigenetic reprogramming of multiple systems, such as the nervous, endocrine and immune systems, during sensitive developmental periods [20]. The second hypothesis suggests that early adversity first reprograms the stress response systems, thereby indirectly affecting various systems including the immune system [21].

The first line of evidence for the causal link between early life adversity (including early maternal deprivation) and the presence of epigenomic changes was established in a number of seminal animal model studies. These models and the corresponding protocols involving laboratory rodents were used primarily to investigate the epigenetic mechanisms modulating the effects of maternal neglect on offspring development: e.g., as a model of maternal deprivation or separation [22, 23], a model of natural variation in maternal care [24, 25], and a model of artificial rearing in the absence of the mother [26]. These studies have revealed that mice and rat pups exposed to maternal separation or raised in “poor” rearing environments exhibited a wide spectrum of detrimental and long-term neurobehavioral effects, manifesting in HPA axis hyperactivity [25, 27], increased anxiety levels [23, 28, 29], learning deficits [30], changes in reward-related behaviors involving alcohol and drug preference [31, 32], and others difficulties. These long-term neurobehavioral effects appear to be modulated by the epigenetic mechanisms that regulate the expression of a number of genes in the brains of neglected offspring, such as the glucocorticoid receptor gene *NR3C1* [25], the corticotrophin-releasing hormone gene *CRH* [27], the arginine vasopressin gene *AVP* [33], and the reelin gene *RELN* [34], as well as many others. Convergently with results obtained by rodent studies, studies utilizing the maternal separation model in rhesus macaques revealed substantial genome-wide differences in DNA methylation in both brain tissue and peripheral T-lymphocytes [35, 36] in monkeys reared without maternal care.

Subsequent studies in human samples also demonstrated that epigenetic states might be changed in response to early adverse experiences, such as child abuse and maltreatment [37, 38], challenged mother-child interactions [39], parental stress [40, 41], and disadvantaged socio-economic position in childhood [42]. Epigenetic changes (currently studied primarily through DNA methylation due to its relative stability and recent technological advances in methylation signal detection and quantification) driven by social environmental factors are system-wide [43] in terms of their impact on both the genome and the organism. Thus, their effects are detectable not only at the level of individual genes [44–46], but also at the level of gene networks that integrate genes involved in the control of key cell signaling pathways [47, 48]. As a consequence of these genome-wide effects, alterations in DNA methylation driven by early social adversities have been detected across various tissues and cells, such as brain tissue [37], buccal cells [46, 49], and peripheral lymphocytes [47, 48]. Moreover, these epigenetic changes might be relatively stable and detectable in adulthood, as found in several retrospective studies of associations between early life experiences and the epigenetic states of adult genomes [37, 48, 50, 51].

Overall, published studies that employed human samples and investigated the mediating role of epigenetic mechanisms in a broad range of negative effects of early adversity are substantially more limited compared to animal research due to both restricted access to suitable biological material and methodological difficulties with recruiting appropriate study cohorts. To our knowledge, to date only few studies have directly focused on the effects of early maternal deprivation in humans in general, and on an extreme case of such deprivation—children's institutionalization—in particular [46, 47, 49, 52]. With the exception of our early study that examined the specificity of DNA methylation states in children of early school age residing in institutions [47], these studies have recruited cohorts of adopted children and adolescents between 12 and 18 years of age, who have experienced institutional care in early childhood but then were integrated into new adoptive families. Using peripheral tissues (blood and buccal cells) and utilizing both candidate genes and genome-wide approaches for DNA methylation profiling (Epigenome-Wide Association Studies, EWAS), these studies have revealed a number of associations between the early experience of institutionalization and epigenetic changes in specific genes, such as the cytochrome P450 gene *CYP2E1* [49] and two stress-related genes: the immunophilin gene *FKBP5* and the serotonin transporter gene *SLC6A4* [46], as well as associations with epigenetic changes in multiple genes controlling core cellular signaling and immune response pathways [47], and neural and developmental pathways [52]. It is necessary to note that only one of these studies [49] investigated the potential involvement of epigenetic modulations driven by early depriving environments in the manifestation of cognitive and behavioral outcomes, specifically long-term behavioral outcomes in adolescents with experience of institutionalization.

Thus, the literature reviewed in this section unequivocally implicates epigenetic changes as modulators of the negative effects of early adversity on development. Nonetheless, to the best of our knowledge, no study has directly investigated the impact of early social deprivation on the epigenome and behavior, and their interplay in young children at a critical stage of development—in infancy and toddlerhood. The remainder of this paper presents the results from a cross-sectional study we conducted to address this gap in the literature. In this study, we compared two groups of children: a group of children who placed into institutional care, and a comparison group of children raised in biological families. This study examined the genome-wide DNA methylation signatures in peripheral blood cells—a tissue that is highly involved in the stress response to internal and external stimuli, and focused on children's adaptive behavior—one of the most vulnerable facets of early development in the context of impact of early adversity—indexed by a comprehensive standardized interview assessment. Therefore, we were not only able to perform cross-group comparisons of genome-wide methylation states but also to further elucidate the modulating role of the epigenome through the examination of the associations between these group-differentiating epigenetic marks and indices of children's emerging adaptive behavior skills.

## Materials and methods

### Participants

The cohorts of participants were ethnically homogeneous and of Eastern Slavic origin. A total of 58 children between the ages of 8 and 35 months participated in the study: 29 children residing in the orphanages (institutional care group, IC) and 29 children being raised in biological families (biological family care group, BFC). The first group, IC children, were recruited from three state-run orphanages (called baby homes) providing institutional care to children up to four years of age in the city of Saint-Petersburg, Russian Federation.

The conditions in the orphanages were relatively homogeneous and regulated by the state. Baby homes in the St. Petersburg region are well equipped to address the children's basic physical and medical needs, and maintain acceptable caregiver to child ratio in care team [6]. Most of the institutionalized children participating in this study (around 70%) were placed into institutional care after birth or within the first year of life. The predominant reason of abandonment was socio-economic—75% of children; 25% of children were removed from their families by the state authorities and placed into institutional care due to child neglect or parental incarceration (S1 Table); children had no records regarding a history of abuse prior to their transition into orphanages.

The second group comprised of children who were being raised in their biological families living in the city of Saint-Petersburg, whose socioeconomic status was characterized as low or low-middle income. Only families with no records or indicators of child abuse and neglect were included in the comparison group. Exclusion criteria in both groups of children were as follows: presence of a history of pregnancy complications, history of mother's heavy drinking and smoking during pregnancy, presence of congenital developmental anomalies, developmental malformations, genetic diseases, and severe systemic disorders.

The IC and BFC groups were matched by the gender and age of the participants (Table 1 and S1 Table): for IC, M_age_ = 19.98 ± 6.77 mos (31% females) and for BFC, M_age_ = 20.89 ± 7.16 mos (38% females). There were no significant group differences with respect to the distributions of children's demographic characteristics (for age, *t*_(56)_ = 0.07; U = 397, Z = 0.35; for gender, χ^2^_(1)_ = 0.999).

**Table 1:** Participant demographics in the two study groups (IC and BFC); Ages are presented separately for the blood draw and behavioral assessment.

Data collected		IC			BFC		
		Females	Males	Total	Females	Males	Total
***Blood draw***							
** N (%)**		9 (31)	20 (69)	29 (100)	11 (38)	18 (62)	29 (100)
** Age, mos**	**Range**	8–33	10–34	8–34	9–30	8–35	8–35
	***M***	21.56	18.7	19.98	20.36	21.22	20.89
	***SD***	7.83	6.02	6.77	6.69	7.41	7.16
***VABS assessment***							
** N (%)**		9 (31)	20 (69)	29 (100)	8 (32)	17 (68)	25 (100)
** Age, mos**	**Range**	12–33	11–34	11–34	10–30	9–37	9–37
	***M***	25.44	20.45	22	21.5	21.82	21.72
	***SD***	6.34	5.67	6.32	5.81	8.60	7.82

### Adaptive behavior skills assessment

To assess children's adaptive functioning, the Vineland Adaptive Behavior Scales-II, VABS-II [53] was utilized. Specifically, we used the caregiver standardized interview form that had been adjusted for young children and had been successfully used with infant and toddler samples in previous studies [54–57]. The semi-structured interview form contained items that evaluated children's functioning in their everyday lives with respect to four specific adaptive behavior domains: Communication, Daily Living Skills, Socialization, and Motor Skills. The Communication domain was assessed with 59 items that indexed receptive and expressive communication; the Daily Living Skills domain was assessed using 43 items indexing children's daily personal, domestic, and community skills; the Socialization domain was assessed using 51 items that index children's interpersonal communication, play and leisure, and coping skills; finally, the Motor Skills domain was assessed using 65 items that probed children's gross as well as fine motor skills.

Due to the specificity of the institutional environment, institutionalized children might have had no opportunity to demonstrate a particular behavior. Consequently, after a careful review of the forms by the research team and the staff of the orphanages, some items, such as "How often does the child pick up the phone?", were omitted to ensure that opportunity bias is minimized and the specifics of the institutional environment are properly taken into account.

We calculated both raw scores and standard scores (hereinafter SS-scores; *M* = 100, *SD* = 15) for each of the four domains; these data are available in S1 Table.

### Procedures

A set of trained interviewers conducted the semi-structured VABS interviews with the children's mothers (for the BFC group) or the caregivers at baby home (for the IC group). For the latter, the caregiver who knew the child best was identified and that caregiver was then interviewed. Four mothers of children from the BFC group refused to provide the detailed interview information, and those data were coded as missing.

All participants underwent a venous blood draw; 0.5–1 ml of blood was collected from each child. For the IC group, the venous blood draws were performed by a medical nurse in the medical offices of the orphanages; for the BFC group, the blood draws were performed in local medical clinics in Saint-Petersburg. The blood draw and the VABS interview dates were scheduled concurrently, but due to study logistics there were gaps; the average gap between the two data collection times was 1.3 months (*SD* = 2.4). Participant demographics broken down by the type of data collected are summarized in Table 1, and individual data are represented in S1 Table.

### Ethical statement

All procedures performed in the study were in accordance with the ethical standards of the institutional and national research committees, and with the 1964 Helsinki declaration and its later amendments or comparable ethical standards. All protocols and procedures of this study were approved by the Saint-Petersburg State University Research Ethics Board. Informed written consents were obtained from the children's primary caregivers: parents for the BFC group and orphanage officials for the IC group.

### DNA methylation analysis

#### Microarray analysis and data processing

Genomic DNA was isolated from peripheral blood using the FlexiGene DNA Kit (Qiagen, Germany) according to the manufacturer's instructions. Approximately 600 ng of DNA was used for bisulfite conversion using the Zymo Research EZ DNA Methylation Kit (Zymo Research, Irvine, CA, USA). To perform genome-wide DNA methylation profiling, the Illumina Infinium MethylationEPIC microarray was used, which covers over 850,000 CpG sites across the human genome regulatory regions. After the bisulfite treatment, 160 ng of DNA was applied to the microarray, as per manufacturer's protocols (Illumina, San Diego, CA, USA).

For the microarray data processing, the *Minfi R* package was used [58]. First, the data were subjected to quality control procedures: the probes with detection *p*-values greater than .05 and probes with missing values were eliminated from the analysis. Next, the probes located on sex chromosomes and probes with known polymorphisms within a CpG site with a minor allele frequency (MAF) of 0.05 or greater (according to the Illumina's manifest) were removed from the analysis. For the remaining probes, the resulting relative methylation level values (beta-values) were in the range between 0 and 1 (fully unmethylated to fully methylated), and underwent further normalization. Both within-array and between-sample normalization was performed using the stratified quantile normalization implemented by the *preprocessQuantile* function in the *Minfi* package.

#### Accession numbers

DNA methylation datasets have been deposed in the NCBI’s Gene Expression Omnibus repository [59] under the accession # GSE118940.

#### Differential methylation analysis

The probes that showed significant group differences (IC vs. BFC) in the average beta-values were identified using the moderated t-test with empirical Bayesian variance method and Benjamini-Hochberg correction to control for multiple testing as implemented in the *Limma R* package [60]. Only epigenome-wide significant (*p*_adj_ < .05) probes were retained, and probes that had a beta-value difference of at least 1.2 fold change between the groups [61] were identified as differentially methylated probes or differentially methylated CpG sites (DMESs).

To control for effects of cellular heterogeneity between samples on the results of differential methylation analysis, we used the corrections implemented in the *FlowSorted*.*Blood*.*450k* package [62] that implements an algorithm [63] utilizing a reference dataset of six different types of white blood cells [64] to estimate cell composition in peripheral blood samples. Multiple linear regression analyses with cell-type counts as predictors of a DMES methylation level were conducted to determine the extent to which the variability in DNA methylation is cell-specific; DMESs that showed a significant association with the cell-type distribution across samples were conservatively eliminated from further analysis.

Genomic annotation of the DMESs was performed using the Illumina's Infinium MethylationEPIC microarray manifest and the UCSC databases [65]. We also relied on a set of analytical tools provided by GeneCards [66], PANTHER [67], g-Profiler [68], and EnrichR [69] to analyze differentially methylated genes (DMEGs) with respect to the biological processes and pathways they are involved in, known molecular functions, and the cellular components they control. Gene-gene interaction networks for DMEGs were examined using the Search Tool for the Retrieval of Interacting Genes/Proteins (STRING) [70]. The false discovery rate-adjusted *p*-values were obtained whenever appropriate to control for multiple testing.

## Results

### Profiles of adaptive behavior skills in IC children vs. BFC children

**Fig 1:**
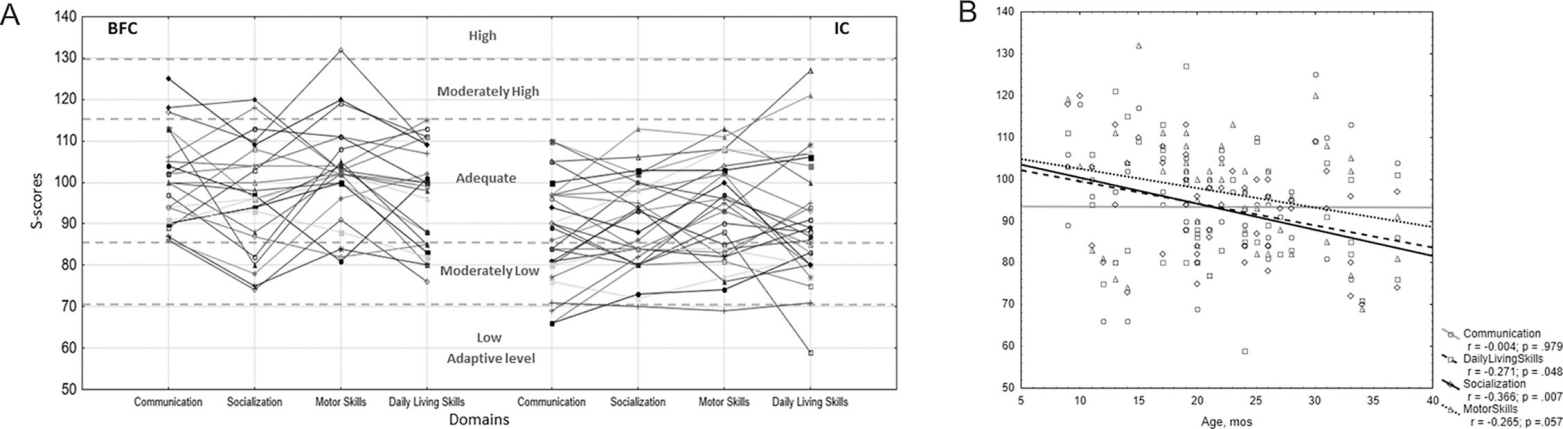
SS-score profiles for the VABS adaptive behavior domains in the IC and BFC groups of children (A); bands of the scores corresponding to a qualitative description of the level of adaptive behavior are marked by dotted lines. Correlations between the VABS domain SS-scores and children's age (B), estimated for the combined sample of 58 children.

Descriptive analyses of the standard scores for the VABS domains revealed that the scores ranged from 88 to 102, and children in both groups demonstrated, on average, an adequate adaptive level in all domains according to the VABS qualitative descriptors [53]. However, relative to the BFC group, the IC group showed a significantly lower average level of adaptive behavior skills in all domains except the Daily Living Skills (Table 2); the largest differences in the average SS-scores were obtained for the Communication and Motor Skills domains, where the effect sizes were estimated at 11.72 and 9.53 standard points, respectively. In addition, the within-group distributions of individual adaptive behavior profiles showed that, in comparison to the BFC group, the IC group had a relatively high frequency of scores in the low-moderately low ranges. With a few exceptions for the Daily Living Skills domain, the children from the IC group generally did not score in the high level of adaptive behavior range (Fig 1A).

**Table 2:** The distribution of SS-scores and Z-scores on four adaptive behavior domains in the IC and BFC groups of children; statistics for between-group comparisons.

Domain	IC	BFC	IC vs. BFC	Welch's *t*-test		*U* test	
	Mean ± SD	Mean ± SD	Difference	*T*	*P*	Z_adj_	*P*
***SS-scores*:**							
** Communication**	88.00 ± 12.38	99.72 ± 10.88	11.72	–3.67	.0006*	–3.19	.0014*
** Socialization**	89.72 ± 10.89	96.68 ± 12.76	6.96	–2.16	.0352*	–1.98	.0477*
** Motor Skills**	92.55 ± 12.09	102.08 ± 11.65	9.53	–2.94	.0049*	–2.52	.0117*
** Daily Living Skills**	90.62 ± 14.87	96.24 ± 11.89	5.62	–1.52	.1354	–1.64	.1007
***Z-scores*:**							
** Communication**	–0.3210 ± 0.8960	0.3724 ± 0.9369		2.78	.0076*	3.04	.0024*
** Socialization**	–0.2456 ± 0.9362	0.2849 ± 0.9505		2.06	.0443*	1.89	.0586
** Motor Skills**	–0.3388 ± 0.9848	0.3930 ± 0.8077		2.96	.0047*	2.59	.0095*
** Daily Living Skills**	–0.1117 ± 1.0662	0.1295 ± 0.8512		0.91	.3677	1.13	.2595

*p < .05; the two-tailed t-test for independent samples was used (df = 52).

Given the absence of norming data for the VABS assessment for children from the general population in Russia, the US national norms were applied to transform the raw scores into standard scores. To examine whether differences in cultural background and in expectations for development rate and adaptive functioning of a child may influence the procedure of scores standardization, we performed a set of linear regression analyses regressing the SS-scores on children's demographic characteristics. We did not observe any significant associations with gender. However, a low but significant negative correlation was obtained between child age and SS-scores for the Socialization and Daily Living Skills domains, *r* = –.366; *p* = .007 and *r* = –. 271; *p* = .048, respectively; similarly, a tendency for negative association between age and SS-scores for the Motor Skills domain, *r* = –.265; *p* = .057 was observed (Fig 1B).

**Fig 2:**
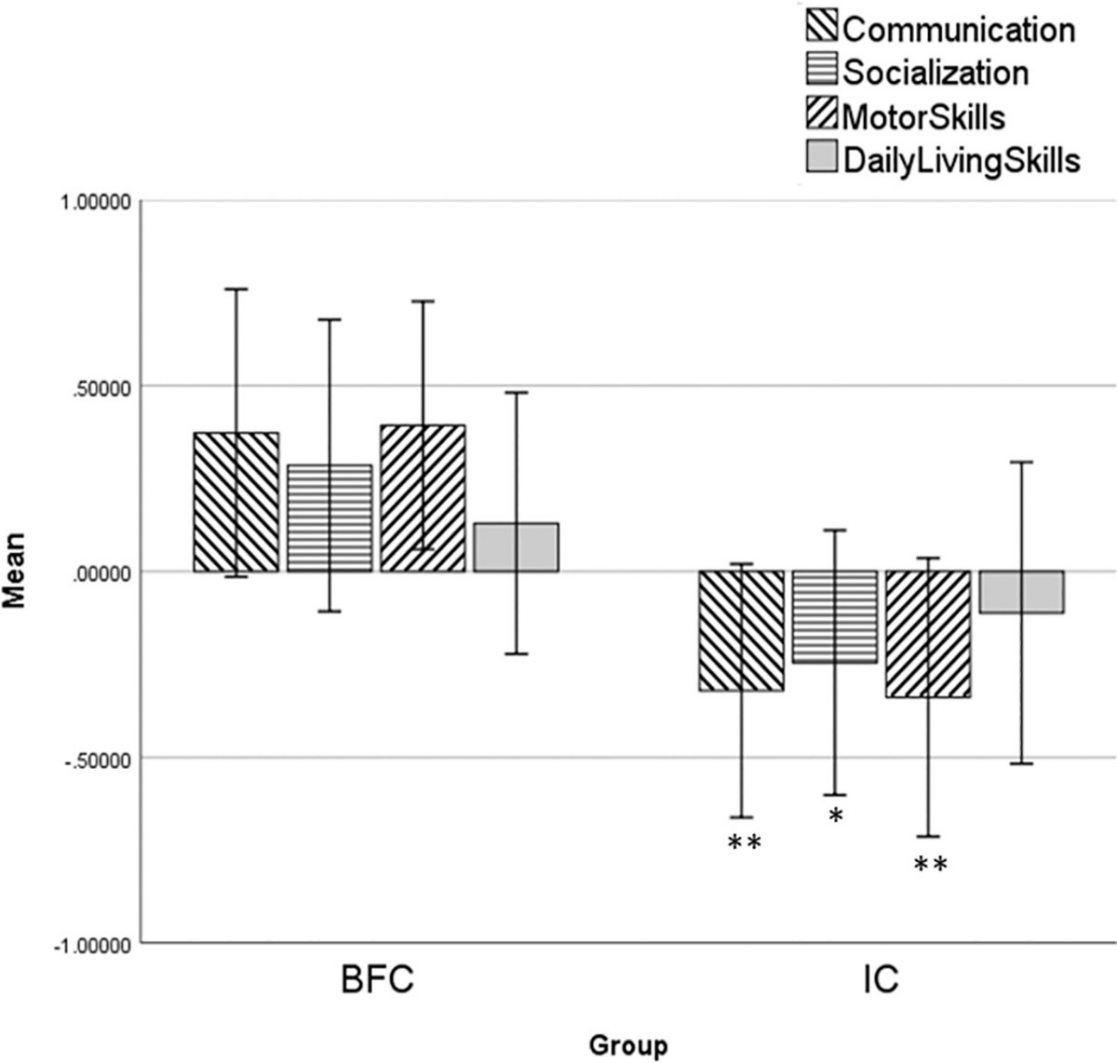
Barplots of average Z-scores for the four VABS adaptive behavior domains. *—denote significant between-group differences at *p < .05 and **p < .01. Means and standard deviations are shown.

To account for these effects in the association analysis, the VABS raw scores in four domains were adjusted for children's demographic characteristics using multiple linear regression; the raw scores were regressed on demographic variables (age, age^2^ and gender), and the standardized residuals were used in further analyses (raw scores and demographics-adjusted Z-scores are available in S2 Table). The distributions of SS-scores and Z-scores and their pairwise intercorrelations are shown in S1 Fig; the determination coefficients for these two types of scores varied from r^2^ = 0.679; *p* < 10^−5^ for Daily Living Skills to r^2^ = 0.764; *p* < 10^−5^ for the Communication domain. The results of the between-group analysis performed using these adjusted Z-scores were consistent with the results obtained for the SS-scores (Table 2; Fig 2).

### Blood cell-type count in IC children vs. BFC children

Given that DNA methylation patterns are cell-type specific, both individual variability and possible systematic group differences in blood cell-type counts may bias the results of differential methylation analyses when comparing the DNA methylation profiles in whole blood. Although the data on blood cell-type counts were not available for the studied sample, we recovered individual cell-type compositions from DNA methylation data [63]; the distribution of six blood cell-types across individuals is represented in S3 Table.

We performed a multivariate analysis of variance (MANOVA) using cell-type distribution as dependent variables and the demographic variables and group status (BFC vs. IC) as independent variables. A significant association of the cell-type composition with children's demographic characteristics (age*gender: F_(12)_ = 2.85; *p* = .003) and a significant difference in cell-type proportion between the IC and BFC groups (F_(6)_ = 3.43; *p* = .008) were detected. A hierarchical clustering analysis showed that individuals from the different groups, IC and BFC, tended to cluster into two distinct clusters based on the blood cell-type distributions (S2 Fig). The results of the between-group comparisons of cell-type composition are shown in Table 3. In comparison to the BFC group, children in the IC group had a higher count of granulocytes and a lower average count of B-cells and CD4^+^ T-lymphocytes. The ratio of T helper cells to cytotoxic T cells (CD4^+^/CD8^+^) was close to the normal ratio (about 2:1) in both groups. In addition, we did not find significant associations between the VABS scores and the blood cell-type composition.

**Table 3:** The distribution of blood cell-types in the IC and BFC groups of children estimated based on DNA methylation data and statistics of the intergroup comparison.

Cell Type	IC group N = 29		BFC Group N = 29		Welch's *t*-test		Mann-Whitney *U* test	
	Mean	SD	Mean	SD	t	*P*	Z_adj_	*p*
**CD4**^**+**^**T-cell**	.242	.072	.283	.066	–2.20	.032*	–2.053	.040*
**CD8**^**+**^**T-cell**	.128	.055	.127	.039	0.11	.916	–0.264	.791
**B-cell**	.143	.043	.187	.051	–3.52	.001*	–3.064	.002*
**NK-cell**	.037	.043	.039	.056	–0.15	.883	0.233	.816
**Monocyte**	.058	.031	.050	.041	0.77	.447	1.182	.237
**Granulocyte**	.397	.105	.318	.078	3.25	.002*	2.924	.003*
**CD4/CD8 ratio**	2.116	0.825	2.389	0.756	–1.32	.193	–1.493	.135

*p < .05; two-tailed tests were performed (df = 56).

### Differential DNA methylation in the genomes of IC children vs. BFC children

#### Differentially methylated CpG sites (DMESs)

As per the Methods section, before the differential methylation analysis was performed, the probes with low signal intensity (n = 9,501) were eliminated; the targets located on sex chromosomes (n = 19,681) and probes containing CpG sites with known common SNPs (MAF > 0.05; n = 11,343) were removed. Methylation levels of 826,311 CpGs that were retained after filtering were subjected to a set of t-tests to detect the CpG sites differentially methylated between the IC and BFC children. After corrections for multiple testing, 3,190 CpGs showed a significant (at a *p*_adj_ < .05) difference in the average methylation levels between the IC and BFC groups. Of those, 256 CpGs showed a between-group difference in the average methylation level of 1.2 and more fold change. Given the cell specificity of DNA methylation profiles and the significant difference in blood cell-type composition obtained for the two comparison groups, a set of multiple linear regressions with cell-type counts as predictors of individual CpG methylation level was conducted. Ninety-two CpG sites whose methylation levels showed a significant association with blood cell-type composition were removed from further analyses, and the remaining 164 CpGs were identified as final differentially methylated CpGs (hereafter DMESs). These 164 DMESs with their relative methylation levels across groups and individuals, as well as the statistics for differential methylation tests, are listed in S4 Table.

**Fig 3:**
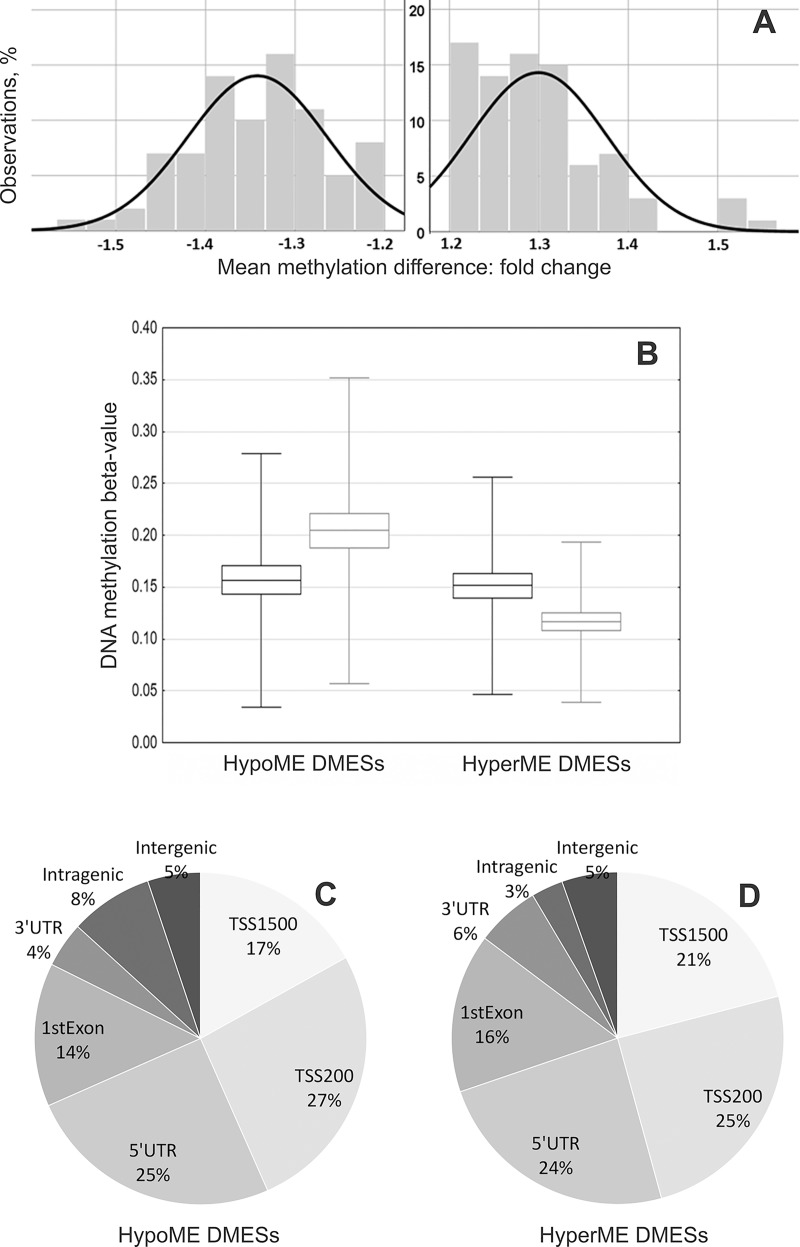
The range of variability for differential methylation indices of the 164 DMESs and their genomic localizations. (A) Distribution of the differential methylation measurement, Fold change; hypermethylation and hypomethylation events in the IC group are shown on the X-axis as positive and negative Fold change values, respectively; the Y-axis represents the number of observations. (B) Box-plots of group differences in the average beta-values for the set of 164 CpG sites—82 hypomethylated (HypoME DMESs) and 82 hypermethylated (HyperME DMESs)—between the IC (black boxes) and BFC (grey boxes) groups of children. Diagrams showing the distribution of hypomethylated (C) and hypermethylated (D) DMESs with respect to their genic region annotation: 1st exon, gene body, 3' or 5' untranslated region (UTR), and promoter-associated region upstream a transcription start site (TSS).

Of those 164 DMESs, 82 CpGs showed an increase in methylation (hypermethylation) and 82 CpGs had a decrease in methylation level (hypomethylation) in the IC group of children, relative to the BFC group. For the vast majority of DMESs, the magnitude of intergroup differences in methylation level varied between 1.2 and 1.4 fold change (Fig 3A). According to the point average estimates and the variance in beta-values, most DMESs were characterized by a generally low methylation level in the genomes of all children (Fig 3B). According to the genome annotation, the DMESs were located predominantly within 1,500 bps of a transcription start site (TSS), and within a 5' untranslated region (5'UTR) (Fig 3C and 3D). Altogether, the genomic annotation of DMESs and their estimated methylation levels (relatively low or moderate methylation) may indicate that between-group methylation variability was mostly associated with the regulatory regions of genes presumably active in the studied tissue.

**Fig 4:**
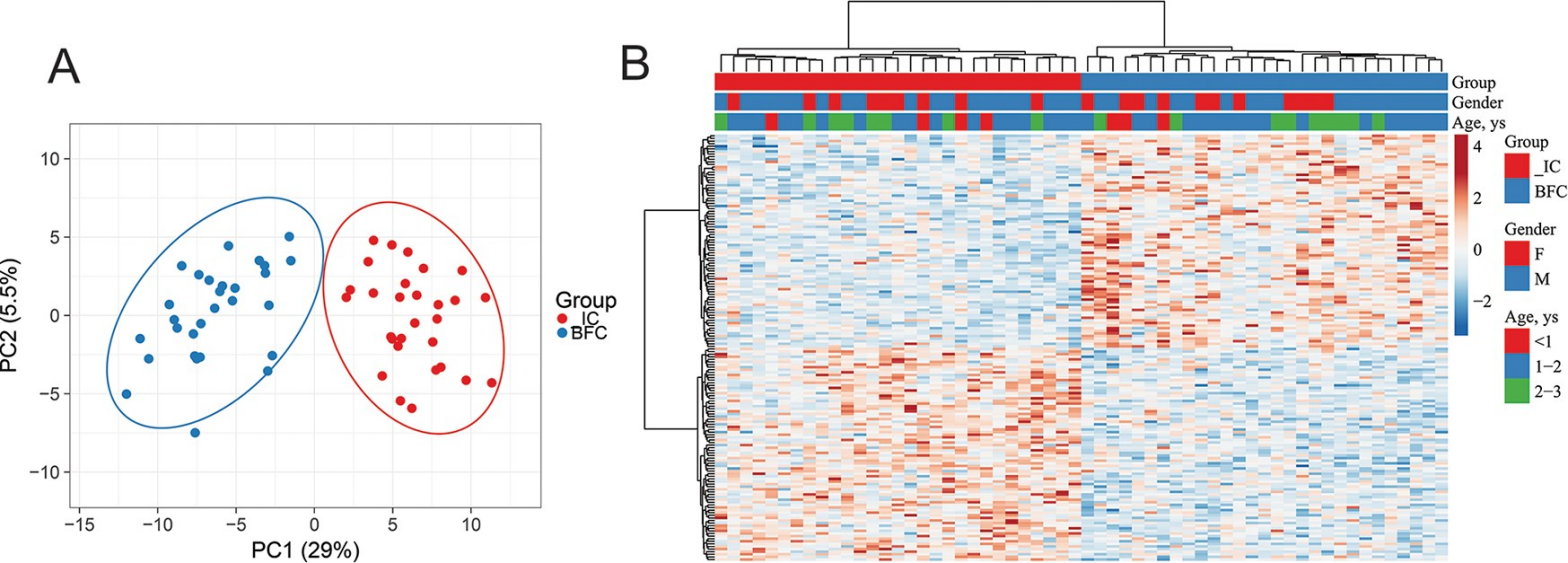
Principal component plot (A) and heatmap (B) demonstrating the clustering of IC and BFC children (columns) based on the methylation levels (beta-values) of the 164 DMESs (rows). The top tracks of the heatmap show individual's group status and demographics.

Principal component analysis (Fig 4A) and hierarchical clustering analysis (Fig 4B) suggested that the resulting 164 DMESs had a high discriminating validity with respect to group status: children clustered into two distinct clusters in accordance to their group status based on methylation profiles for these DMESs. Demographic variables did not contribute to the main clustering structure; however, children of same gender and similar age tend to cluster together in smaller subclusters along the classification tree.

We performed a set of multiple regression analyses with demographic variables as predictors of a CpG methylation level and found that about 10% of 164 DMESs (k = 17) had a low or moderate but significant (at a *p* < .05) association with either child's age or gender. Since the IC and BFC groups were matched on demographics, these associations were unlikely to affect the results of between-group comparisons, yet they may influence the results of the association analysis between the methylation states and the VABS adaptive behavior measures. Therefore, we converted the beta-values using an approach similar to the one we used to transform the VABS scores: beta-values were regressed on demographic variables, age and gender. Both types of methylation measurements, beta-values and adjusted on demographics z-values, were highly correlated, with Pearson's *r* ranging from 0.867 to 0.999; *r*_avg_ = 0.982 (S3 Fig).

#### Differentially methylated genes (DMEGs)

Genomic annotation revealed that most of 164 DMESs were located within CpG islands (CGI) in regulatory regions of genes (Fig 3C; S4 Table and S5 Table). Cumulatively, the DMESs were annotated to 172 such genes (DMEGs). All DMEGs were represented by a single DMES except for two genes, which included two CpG sites each (S5 Table): *PCBP3* (Poly(rC) binding protein 3) involved in posttranscriptional activities and mRNA metabolic processes and *SOCS3* (Suppressor of cytokine signaling 3).

**Fig 5:**
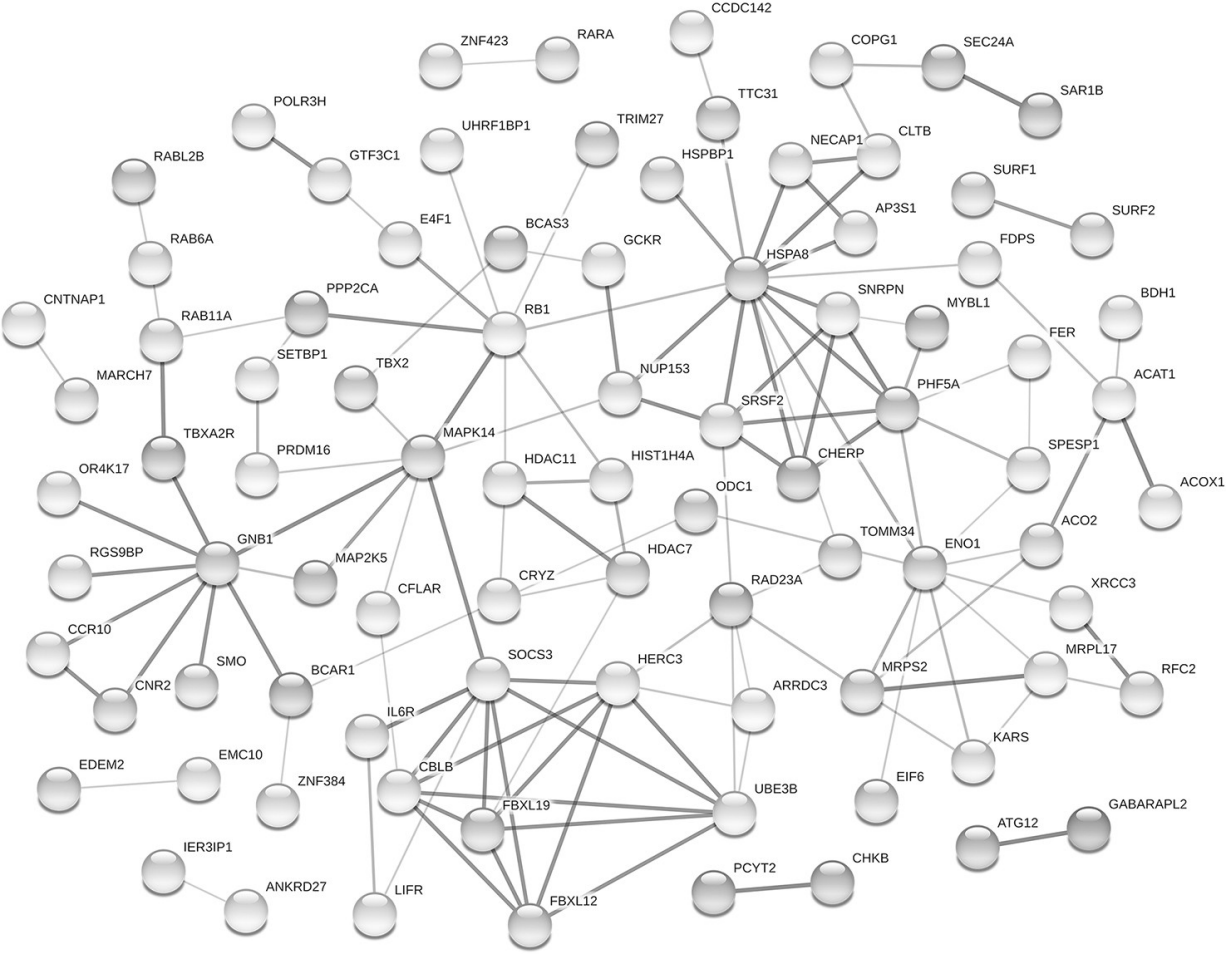
The STRING network representing known gene-gene interactions for 172 genes differentially methylated in the IC group. The nodes represent genes; only connected nodes are shown (k = 87). The color saturation of edges corresponds to the confidence score of the functional association; medium (> .400) and high (> .700) scores are denoted by light grey and dark grey, respectively.

The enrichment analysis based on the DMEGs did not reveal a significant overrepresentation of particular molecular functions or biological pathways. To increase the power of pathway enrichment analysis, an additional overrepresentation test was performed for the curated list of 87 DMEGs with established gene-gene interactions, which was derived using the STRINGdb analytical resources [70] based on cumulative data on gene co-expression, molecular interaction, and joint involvement in a pathway or phenotype (Fig 5). According to the STRING network, six genes had central position (re: multiplicity and statistical validity of gene-gene interactions) in the network: *MAPK14*, *ENO1*, *GNB1*, *RB1*, *SOCS3*, and *HSPA8*. Five of them (except the *MAPK14*) were found to be downmethylated in the IC group; and all of them involved in the control of crucial pathways in immune cells**'** function with a special focus on cytokine signaling.

Thus, *MAPK14* encodes a MAP-kinase, which, when activated by environmental factors and anti-inflammatory cytokines, plays a key role as a regulator of biochemical signals involved in a spectrum of biological processes, such as cell proliferation and differentiation, transcription regulation, and cell and organism development. The *RB1* gene is a key regulator of cell division; this DNA binding transcription factor is involved in heterochromatin formation by maintaining overall chromatin structure through the stabilization of histone methylation. The *GNB1* gene encodes a guanine nucleotide-binding protein (G protein) that is implicated as a modulator or transducer in various transmembrane signaling systems. The multifunctional enolase enzyme encoded by *ENO1* is involved in glycolysis; it plays an important role in such biological processes as growth control, tolerance to hypoxia, and allergic reactions. The product of the *SOCS3* gene is a protein that functions as a component of a complex that mediates the ubiquitination and subsequent proteasomal degradation and regulates cytokine signaling in the immune system. The *HSPA8* encodes a protein of the heat shock family, which expresses in response to cellular stress due to heat, toxic chemicals, and diseases. HSPA8 functions as a chaperone; it is involved in the restoration of protein structures, in the formation and dissociation of protein complexes. In the immune system, it binds bacterial lipopolysaccharide and mediates inflammatory response, including TNF secretion by monocytes; also, among its related pathways in the immune system is cytokine signaling.

With the exception of *ENO1*, six highlighted genes mapped onto a set of functional groups and pathways identified as overrepresented based on the list of 87 DMEGs (Table 4). The pathway analysis uncovered a statistically significant enrichment of the pathways involved in cellular response to external stimuli and cellular response to stress, including the stress-response mediated by p53 transcription factor, which, when activated through ubiquitination, initiates a program of cell cycle arrest, cellular senescence or apoptosis (Table 4). In addition, the list of 87 DMEGs was enriched for pathways involved in posttranslational protein modifications (especially ubiquitination), intracellular transport (especially vesicle-mediated transport occurring via the endoplasmic reticulum and Golgi transport), and cellular signaling. Among the overrepresented cellular signaling systems, three particularly noteworthy: (1) signal amplification or thromboxane A2 receptor activation leading to platelet activation, signaling and aggregation; (2) interleukin-6 signaling and (3) gastrin and cholecystokinin receptors (CCKR) mediated signaling network, which provoke a broad range of cellular and physiological responses, regulating cell growth, proliferation, survival, and differentiation.

**Table 4:** Summary of the results from the pathway overrepresentation analyses based on the 87 DMEGs in the STRING network: the Gene Ontology (GO), KEGG (has), Reactome (HSA), and the Panther (P) databases; core genes in the network are shown in bold.

Pathways		Genes	*p*_adj_
GO:0009605	Response to external stimulus	*CCR10; CFLAR; ATG12; FER; ACKR2; GABARAPL2*	3.86E-02
HSA2262752	Cellular responses to stress	***RB1****; GABARAPL2;* ***HSPA8****; HIST1H4A; NUP153;* ***MAPK14****; ATG12*	4.96E-02
P04398	p53 pathway feedback loops	*PPP2CA;* ***RB1***; ***MAPK14***	1.93E-02
HSA6783589	Interleukin-6 family signaling	***SOCS3***; ***MAPK14****; LIFR; IL6R*	4.00E-02
P06959	CCKR signaling map ST	*ODC1;* ***GNB1***; ***MAPK14****; MAPK2K5; BCAR1; ACAT1; HDAC7*	9.36E-04
HSA392518	Signal amplification	*TBXA2R;* ***MAPK14***; ***GNB1****; RAB11A*	4.00E-02
GO:0046907	Intracellular transport	*TOMM34; RABL2B; GCKR; EDEM2; RAB11A; ANKRD27;* ***HSPA8***; ***MAPK14****; SEC24A; CBLB; NUP153; SMO; IER3IP1; BCAS3; FER; SAR1B; SRSF2; CLTB; RAB6A; AP3S1; TRIM27; EIF6*	3.73E-03
GO:0006886	Intracellular protein transport	*NUP153; ACOX1; RAB6A; EIF6; RABL2B; CLTB; CBLB; SEC24A; SAR1B; TOMM34; SRSF2;* ***HSPA8****; COPG1; AP3S1; GCKR; RAB11A*	9.80E-03
GO:0016192	Vesicle-mediated transport	*IER3IP1; SPESP1; RAB6A; ANKRD27; RABL2B; CLTB; BCAS3; RAB11A; SEC24A; SAR1B; FER;* ***HSPA8****; AP3S1; COPG1; NECAP1*	1.93E-04
HSA199991	Membrane trafficking	***HSPA8****; NECAP1; SAR1B; CLTB; AP3S1; RAB6A; RAB11A*	4.00E-02
has04141	Protein processing in ER	***HSPA8****; HSPBP1; SEC24A; SARB1B; EDEM2; RAD23A*	1.45E-02
HSA74182	Ketone body metabolism	*BDH1; ACAT1*	4.00E-02

#### Methylation of the glucocorticoid receptor gene NR3C1

**Fig 6:**
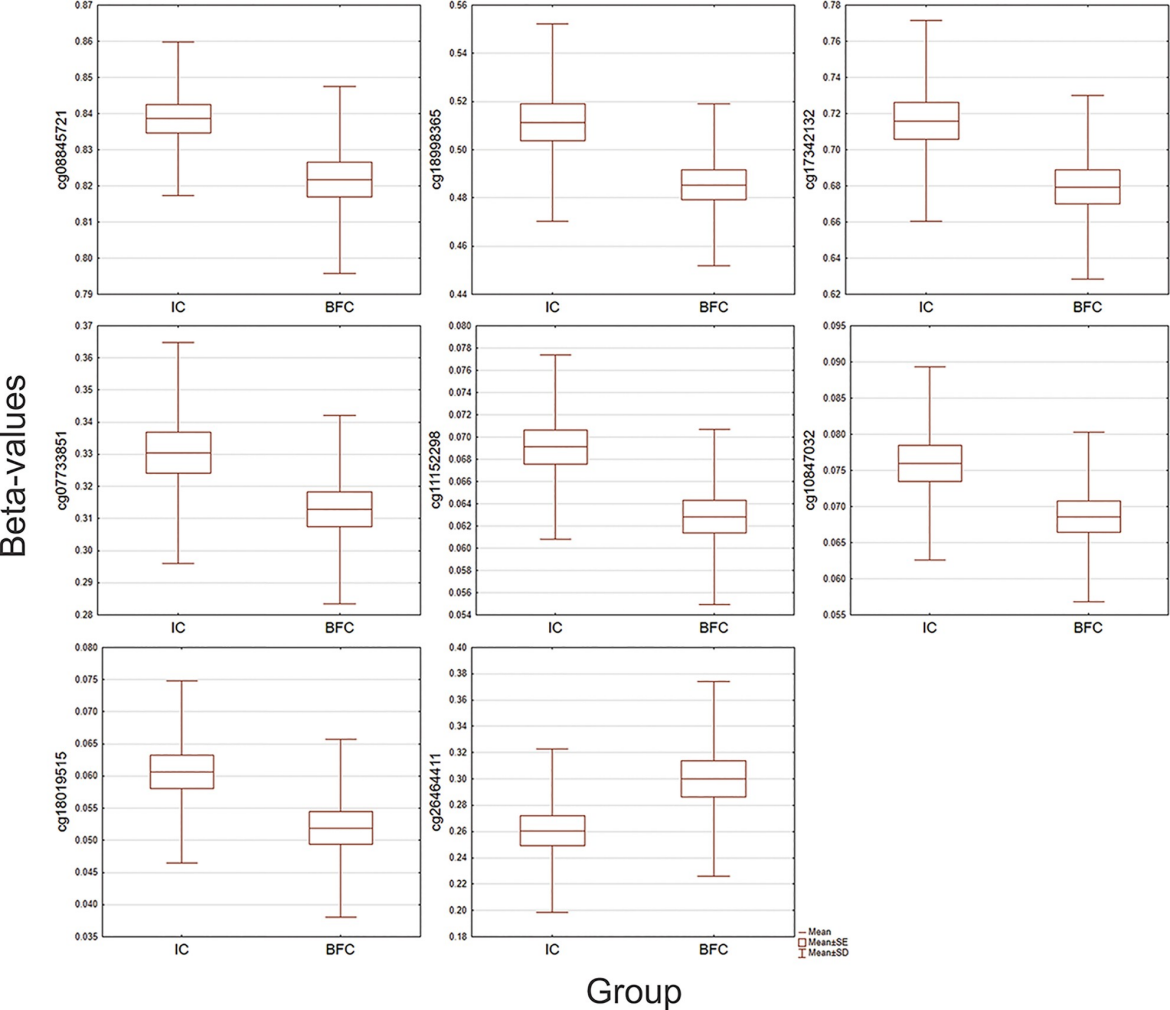
Boxplot graphs illustrating group differences in methylation levels (beta values) for eight CpG sites located within the CGI (chr5:142782071–142785071) associated with the promoter of the *NR3C1* (Glucocorticoid Nuclear Receptor Variant 1) gene, which showed a nominally significant (at a p < .05) difference in methylation between the group of institutionalized children (IC) and the group of children raised by their biological families (BFC).

Early life adversity has been linked with hypermethylation events in the CpG island (CGI; chr5:142782071–142785071) of approximately 3kb related to the *NR3C1* promoter, which have been detected in different tissues, including the CNS and peripheral tissues such as blood and saliva [71, 72]. The MethylationEPIC array utilized in this study contains probes for 42 CpGs located within this CGI and its flanking regions (shelves and shores). Our EWAS did not reveal a statistically significant difference in the *NR3C1* methylation between the IC and BFC groups after the multiple testing correction. However, eight of these 42 CpGs showed a significant (at a nominal *p* < 0.05) difference in the average methylation level between the groups. Specifically, one CpG was hypomethylated and seven CpGs were hypermethylated in the IC group (Fig 6; S6 Table). It is necessary to note, we did not observe a significant association of the methylation profile of the *NR3C1* promoter CGIs neither with the children's VABS adaptive behavior scores, nor with the duration of institutionalization.

### DNA methylation and children's adaptive behavior

**Fig 7:**
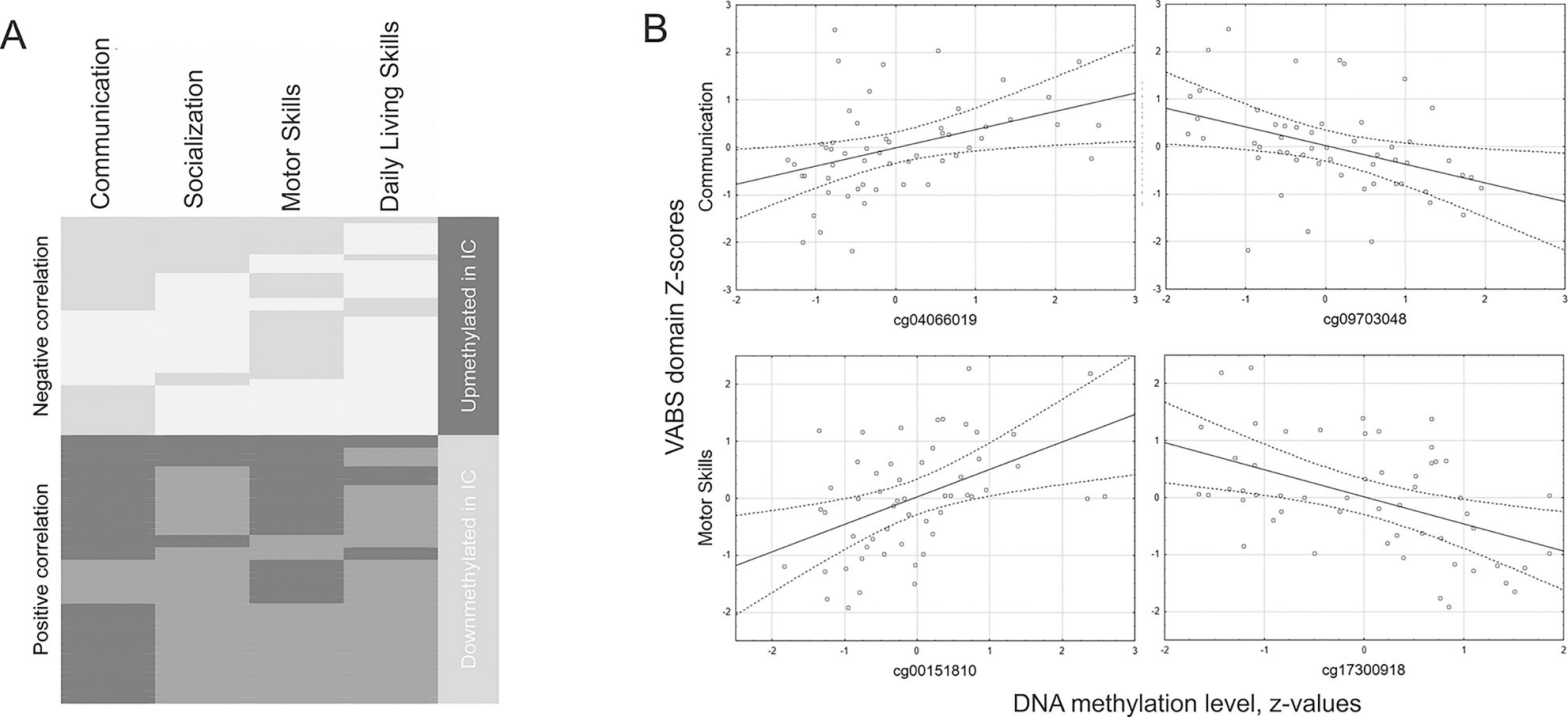
Associations between Z-scores on the four VABS domains and the methylation levels (z-values) of CpG sites differentially methylated in IC children. (A) Clustergram depicting correlations for 78 DMESs (rows) that showed a significant (*p*_nominal_ < 0.05) association with at least one VABS domain. Negative and positive associations are shown in light grey and dark grey, respectively; the color saturation denotes *p*-values—lighter for a *p*_nominal_ > .05 and darker for *p*_nominal_ < .05. The last column encodes the direction of methylation level difference (up- and downmethylation) in the IC group. (B) Scatterplots representing correlations between CpGs methylation levels and VABS scores; four significant (*p*_adj_ < .05) associations are shown.

To explore the associations between the methylation states of DMESs and the levels of child adaptive behavior and everyday skills, we performed the Spearman's rank correlation analysis between the methylation z-values of the 164 DMESs and Z-scores on the four VABS behavior domains in the combined sample of 58 children. Of 164 DMESs, 78 CpGs showed a significant (at a *p*_nominal_ < 0.05) correlation with at least one of the four VABS domains (Fig 7A; S7 Table). The associations were highly concordant in terms of directionality (either positive or negative correlations) across all four domains; the Communication and the Motor Skills domains showed the greatest number of significant correlations with CpGs methylation levels. Notably, the methylation states of these 78 DMESs in IC children were opposite to those that seem to be “beneficial” for the development of adaptive behavior and everyday skills. Specifically, CpG sites that showed a positive association with adaptive behavior in the combined sample were hypomethylated in IC children, and vice versa (Fig 7A).

After the correction for multiple comparisons, four associations remained significant (at a *p*_adj_ < 0.05) for the Communication and Motor Skills domains (Fig 7B). Specifically, the Communication scores had a positive association with the methylation level of cg04066019 located within 5'UTR of the *RFTN1* gene and a negative association with the methylation level of cg09703048 located within the region of 1,500 nt upstream of TSS (transcription start site) of the *OR4K17* gene. *RFTN1* (Raftlin, Lipid Raft Linker 1) is a key regulator of the formation and maintenance of lipid rafts involved in many signal transduction processes; in the context of blood tissue this protein is involved in B-cell and T-cell-mediated immune responses. *OR4K17* encodes an olfactory receptor, which together with other members of olfactory receptors family initiates a neuronal response that triggers smell perception.

The VABS Motor Skills domain scores showed a significant positive correlation with the methylation level of cg00151810 and a negative correlation with cg17300918 (Fig 7B). The former CpG is localized in the promoter (TSS200) of the *BCAR1* gene (Breast Cancer Anti-Estrogen Resistance 1), which encodes a docking protein playing a central coordinating role for signaling related to cell adhesion. The latter CpG is localized in the promoter of the *LTBP1* gene that encodes the Latent Transforming Growth Factor Beta (TGFB) binding protein 1, which plays a critical role in controlling and directing the activity of TGFB1.

### Effects of the institutionalization duration on children's behavior and epigenome

To explore the potential cumulative effect of institutionalization on children's adaptive behavior and epigenomic states, a set of association analyses was performed. A child exposure to institutionalization (duration in months) was considered as a predictor of the child adaptive level and epigenetic status. The association analyses were carried out separately: (1) at the level of individual variability of scores on adaptive domains and methylation measurements of 164 DMESs within IC group and (2) at the level of general behavioral and epigenetic differences between IC and BFC children. For the former, we estimated partial correlations between a CpG methylation level and the duration of exposure in months controlling for gender and the age of child at placement into institutional care. For the latter, we calculated the Euclidean distances between individual profiles in the IC group and the averaged profile for the BFC group (hereinafter “normative” profile) as a metric of the distance of individual behavior profiles (hereinafter D_VABS_) or DNA methylation profiles (hereinafter D_EPI_) from the normative profile.

Summarizing the results of the first analysis, it is necessary to note that our power to detect a statistically significant effect of the duration of institutionalization on adaptive behavior scores and methylation levels of particular CpGs was limited due to the small sample size. The scores for the four VABS domains showed low in magnitude and non-significant but consistently negative associations with institutionalization duration: correlation coefficients varied from –0.053 to –0.18, *p* > 0.1, for the Motor Skills and Communication domain, respectively. In turn, methylation levels of seven CpG sites showed an association (at a *p*_nominal_ < .05) with the duration of institutionalization; among them, three CpGs were localized in a gene promoter region, TSS1500 (Table 5). The duration of institutionalization was negatively associated with the methylation level of CpGs in promoters of the *ACAT1* gene*—*a mitochondrial acetyltransferase that plays a major role in ketone body metabolism, and the *RB1* gene—a key regulator of entry into cell division and a transcription factor involved in heterochromatin formation. A positive association, or an increase in promoter methylation with the increase in exposure duration, was obtained for *CNTNAP1*, which encodes a contactin-associated protein involved in the signaling between axons and myelinating glial cells in the brain.

**Table 5:** List of CpGs that showed a significant (at a p_nominal_ < .05) correlation with the duration of institutionalization in the cohort of IC children.

CpG Index	Correlation coefficient	*p*-value	Gene Name (Region)	Gene Function	IC DiffME*
cg00372669	–0.307	0.013	*NDST2* (5'UTR)	Bifunctional heparan sulfate N-deacetylase/N-sulfotransferase 2	Down
cg00385769	–0.458	0.014	*C6orf47* (5'UTR)	Uncharacterized protein C6orf47	Down
cg08360253	0.386	0.042	*PPP2CA* (3'UTR)	Protein Phosphatase 2 Catalytic Subunit Alpha	Up
cg13363025	0.402	0.034	*HYAL4* (Body)	Hyaluronidase-4	Up
cg14994056	–0.382	0.045	*ACAT1* (TSS1500)	Acetyl-CoA acetyltransferase, mitochondrial	Down
cg22766818	–0.403	0.033	*RB1* (TSS1500)	Retinoblastoma-associated protein	Down
cg24642546	0.373	0.049	*CNTNAP1* (TSS1500)	Contactin-associated protein 1	Up

*IC DiffME indicates the difference (Up- or Down-methylation) in the average methylation levels in the IC group compared to the BFC group.

**Fig 8:**
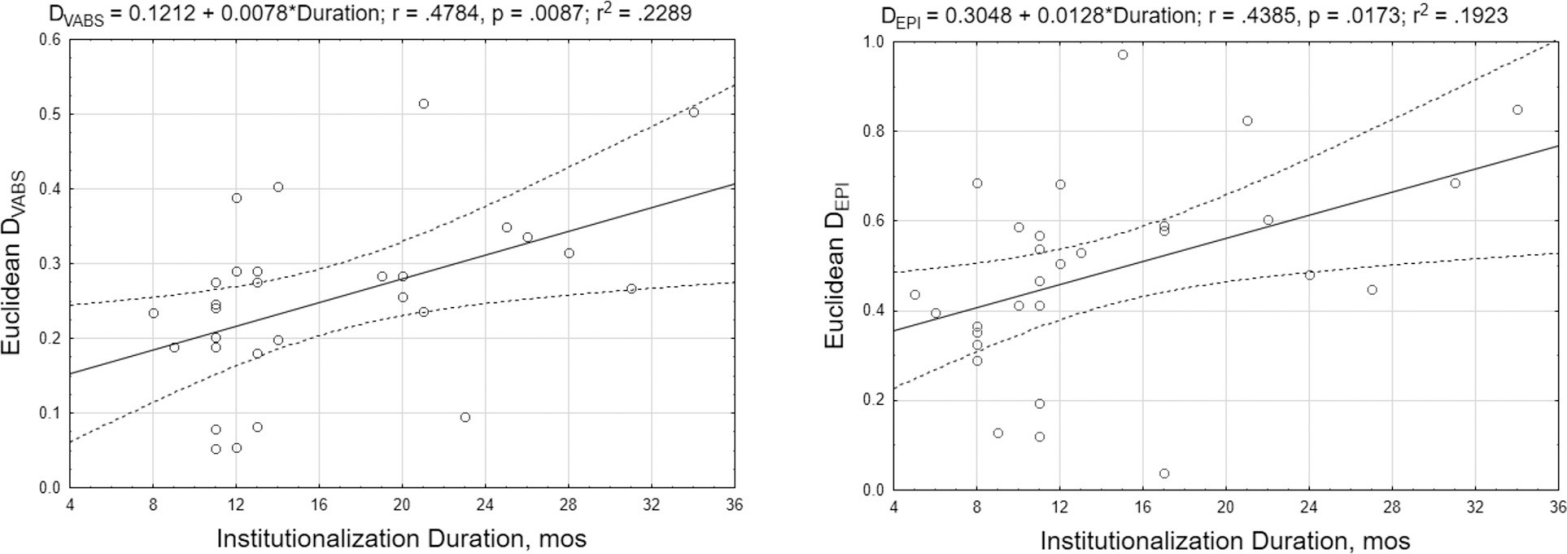
Scatterplots representing the associations between the duration of institutionalization and the distance (Euclidean D in the range from 0 to 1) of IC children's behavioral profiles (D_VABS_ ; left plot) and DNA methylation profiles (D_EPI_; right plot) from the average profile in the BFC group.

**Fig 9:**
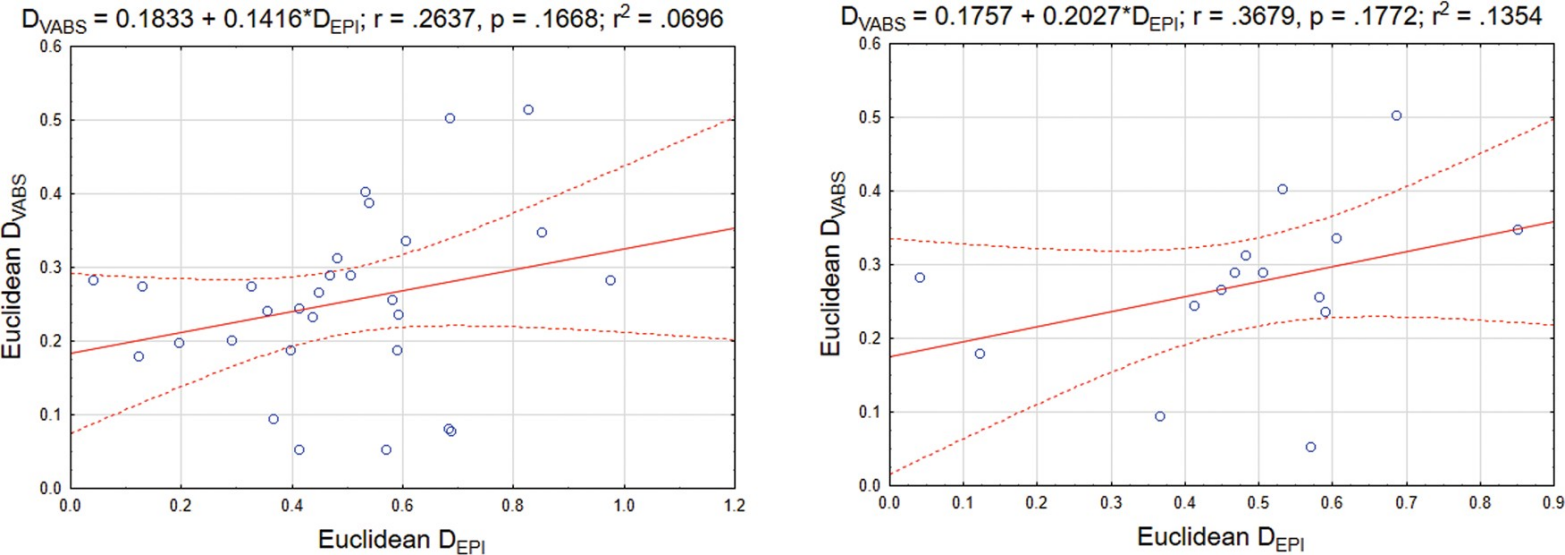
Scatterplot graphs depicting the associations between the index of the deviation (from the averaged profile of children in the BFC group) of the VABS behavior profiles (D_VABS_; y-axis) and DNA methylation profiles (D_EPI_; x-axis) in the group of IC children. Two graphs are presented: data from the total sample of IC children (N = 29; left plot) and for the subgroup of children who have been placed into institutional care after birth (N = 15; right plot).

The second analysis, focused on the effects of institutionalization duration on general behavioral and epigenetic differences between the IC and BFC groups, revealed a significant association between the time children had spent in orphanage and the distances of their behavioral and epigenetic profiles from the “normative” profile; *r* = .4784; *p* = .0087 and *r* = .4385; *p* = .0173, respectively (Fig 8). In turn, the distance of behavioral and epigenetic profiles of IC children from the “normative BFC profile” showed a tendency for positive association. Thus, despite a lack of statistical significance, the correlation between D_EPI_ and D_VABS_ was positive (*r* = .2637; ns) and even larger in magnitude in the subgroup of children (n = 15) who have been placed into the institutional care after birth (*r* = .3679; ns). The analyses revealed that D_EPI_ and D_VABS_ shared approximately 7–14% of variance with *R*^2^ = 0.0696 and 0.1354 for the entire cohort of 29 IC children and for the subcohort of 15 children institutionalized at birth, respectively (Fig 9), thereby directly linking group differences in adaptive behavior with those in DNA methylation levels.

## Discussion

Our study aimed to investigate the impact of early institutionalization, as a special case of social-emotional deprivation, on children's epigenome and behavior, and their interplay. Using a cross-sectional design and well-established instruments and analytical approaches, we compared two groups of children—children raised in institutional care and their age/gender peers raised in biological families—on a set of adaptive behavior skill indices, as well as on a set of epigenome-wide DNA methylation differences.

In contrast to recent studies exploring residual epigenomic footprints of early institutional experience in the genomes of adolescents [46, 49, 52], this study focused on (1) current epigenetic states of children residing in orphanages, who were (2) in the age range characterized by intense sensitive periods of postnatal life—infancy and toddlerhood, which are crucial for the immune system maturation [73] and for a vast array of developmental processes and facets [74–77]. Another crucial element of our study was the examination of the potential involvement of epigenetic modifications in the development of adaptive behavioral skills, indexed by a standardized semi-structured caregiver interview obtained for institutionalized children, who are known to be manifesting multiple developmental delays and deficits, including a deficit in the development of adaptive skills [5–7].

Elevated prevalence of developmental delays and deficits among institutionalized children may be due to the overrepresentation of children with various developmental diseases and disorders in this specific cohort. It has been reported that about 25% of institutionalized children have disabilities due to congenital anomalies and birth defects, genetic abnormalities and nervous system disorders [5, 6]. This prevalence increase may be driven by both (1) pronounced background of socio-economic and health risks in families where parents have been deprived of parental rights or have made their own decision to place their child into an institutional care, and (2) the elevated abandonment risk for children who are born with developmental delays and/or special needs.

Given the families' social background and the specificity of institutionalized cohort, we enrolled in the study only those children who did not have a documented medical history of prenatal burdens, developmental malformations and severe systemic disorders, to maximally exclude the factors that could bias the results of the study focused on the effects of institutionalization on typical child development. Despite taking these precautions, we are mindful of other possible confounding factors that were not recorded in a children medical data but may nonetheless contribute to the differences (both behavioral and epigenetic) between the institutionalized children and children raised in enriched biological family environments. These include, for example, maternal depression during the pregnancy and/or the stress associated with the need to make the decision to abandon the child, early adversity that the child could experience in infancy before he/she was removed from his family. Correspondingly, we are cautious in interpreting group differences between institution-reared and biological family-reared children with respect to the causal role of the institutional environment alone.

### Institution-reared children significantly differ from their biological family-reared peers regarding their adaptive skills and epigenomic statuses

In concordance with previously reported findings connecting institutional environments with subsequent negative developmental outcomes, the results of our comparative study provide strong evidence that children residing in orphanages significantly differ from their biological family-reared peers regarding both their biological (i.e., indexed by DNA methylation) and behavioral (i.e., indexed by the VABS assessment) characteristics. Briefly, the results of these between-group comparisons can be grouped into three observations.

First, we found significant between-group differences in the level of children's adaptive skills assessed as per the VABS [53]. Prior research suggests that institution-reared infants and toddlers show delayed motor skill development and poorer social skills [5, 6], paralleled by similar findings in cohorts of post-institutionalized adopted children of older age [78, 79]. The consensus in the literature is that these developmental delays are related to the depriving nature of institutional environments that do not provide children with appropriate age-expectant opportunities for relevant skill development. In our study based on a preselected sample of kids without a serious diagnosis and/or developmental dysfunction, we did not observe significant behavioral deficits or delays in the development of adaptive skills in the institutionalized children; most of them demonstrated an adequate adaptive level in all domains. However, in comparison with biological family-reared children, the cohort of institutionalized children on average displayed lower levels of adaptive skills across multiple indexed domains. Specifically, a significant difference was observed for the development of communication, social and motor skills.

Second, we observed a significant difference in immune-phenotypes between the groups of children raised in institutional care vs. children raised in biological families. For this type of analysis, we estimated the individual distribution of white blood cells, using an algorithm allowing the back-prediction of cell-type distribution based on the cell-specificity of DNA methylation profiles [63]. The goal of this analysis was twofold: the recovered cell-type compositions were used as a proxy for immune-phenotypes in the between-group analysis of children's immune statuses and for the adjustment of DNA methylation profiles for cell-type heterogeneity.

In comparison to children raised in biological families, institution-reared children had a higher average count of granulocytes that may indicate a higher risk of allergic reactions, infections and autoimmune diseases. Previously, elevated levels of HSV-specific (herpes simplex virus) antibodies were found in saliva specimens of youth with an experience of early institutionalization and physical abuse; given the similar prevalence of HSV infection across the groups, including the control group, this altered antibody profile was proposed to be linked with the failure of cellular immune processes to limit viral reactivation in youth with negative experiences [80]. In addition, the cohort of institutionalized children had a lower average count of B-cells and T-helpers (CD4^+^) that, in turn, may indicate a deficit in the adaptive immune system. Similar effects, namely a significantly smaller percentage of CD4^+^ T-cells and B-cells in the blood cell-type composition predicted based on whole-blood DNA methylation profiling, have been observed in a sample of adopted youth with a history of early institutionalization in a recent study [52]. Perhaps due to small sample size, we did not observe an association between children's adaptive behavior ratings and their inferred blood cell-type composition, which we considered in this type of analysis as a surrogate measure of a child’s immune status and which, as we expected, might be associated with development.

We would like to note that the hypothesis that early adverse experiences may be associated with the peripheral leukocytes count, and that this alteration might be a long-term physiological effect, has been previously supported by the results of a large-scale population-based cohort study in adults [81]. Thus, the results of our study, first, provide additional evidence in support of the hypothesis that postnatal adverse experiences may significantly affect immune function [82, 83]; and second, they point to the need to take into account the prevalence of lymphocyte subpopulations in the analyses comparing whole-blood DNA methylation profiles between the groups with known or expected differences in immune-phenotypes.

Third, we found significant differences in DNA methylation between the two comparison groups of children. Group-specific DNA methylation profiles were defined based on 164 differentially methylated CpG sites whose methylation levels were not related to either blood cell-types counts or children's demographic variables. These methylation sites were associated with 172 genes. Subsequent gene-network and gene function enrichment analyses revealed several key genes and biological pathways, whose methylation patterns had significant differences in the genomes of institutionalized children. Among these, several core multifunctional genes might be highlighted: *RB1*, *GNB1*, *ENO1*, *SOCS3*, *HSPA8*, and *MAPK14*; and all of them (except the *MAPK14*) were found to be downmethylated in the IC group. Taken together, the direction of differential methylation changes in these core genes between the groups and the functions of these genes in the immune system indicate that institutionalized children may display a different pattern of the immune system activity that seems to be related to cytokine activation, a known immune response to toxic stress [84]. Convergently, elevated level of circulating cytokines has been identified as one of the reliable immune markers associated with adverse experiences in childhood [85, 86]. The pathway enrichment analysis of the list of differentially methylated genes also provided additional evidence regarding the significant perturbation in the epigenetic regulation of the immune system function in children raised in institutional care. Specifically, we established the significant enrichment of pathways linked with cellular response to stress and external stimuli, cellular signaling pathways involved in the regulation of cellular growth, proliferation, survival and differentiation (such as interleukin-6 signaling and gastrin and cholecystokinin receptors mediated signaling), and pathways related to membrane trafficking and vesicle-mediated transport, which, in turn, are associated with intercellular signaling and endocytic pathways of immune cells.

It should be noted, that we did not observe a stringent overlapping of the differentially methylated events (particular CpG sites or genic regions) found in the genomes of institutionalized toddlers with those previously reported in studies focused on DNA methylation profiling in youth and adults with an experience of institutionalization [47, 49, 52], or with other negative early experiences, such as child abuse [48, 87] and early socio-economic disadvantages [42]. This might be partly related to the differences in the ages of studied cohorts (youth and adults vs. infants and toddlers in this study), which represent different stages of development and the immune system maturation. However, we found multiple parallels between our findings and those reported in the literature. Thus, alterations in DNA methylation in MAPK signaling has been reported in association with disadvantaged socio-economic position in childhood [42]. Transcriptional alterations in genes involved in MAPK signaling pathway, together with the genes controlling cytokine-cytokine receptor interaction pathways, have also been detected in the monocytes of adults with a history of childhood adversity [88]. Moreover, the immune response and cell signaling have been highlighted as the pathways that undergo the most significant epigenetic perturbations in the genomes of 7–10 years aged children reared in institutional settings [47].

A targeted examination of the results from the differential methylation analysis did not reveal a significant difference in methylation of the glucocorticoid receptor gene *NR3C1* between comparison groups of children. Since the publication of the animal study that has revealed an epigenetic modification of the *NR3C1* as an indicator of differential level of maternal rearing behavior [25], a significant number of studies have focused on the epigenetic modulations of the *NR3C1* in humans in the context of their association with various psychopathology, stress reactivity and early stress conditions [71, 72]. Early life adversity has been frequently associated with hypermethylation events in a CpG island related to the *NR3C1* promoter. In our sample, we observed nominally significant between-group differences in the methylation levels of eight CpGs within the CGI in *NR3C1* promoter, seven of which were upmethylated in the cohort of institutionalized children. A similar observation of hypermethylation events in the *NR3C1* promoter has been made in a study of adults with experience of early institutionalization in which candidate gene pyrosequencing was utilized [89].

### Epigenetic marks associated with the development of children's adaptive behavior skills

In our study, we attempted to explore the potential associations of specific epigenetic states distinguishing institutionalized children from their biological family-reared peers with the development of children's adaptive behavior. We found that almost 50% of the CpGs differentially methylated in the institutionalized children showed a nominally significant association with the level of children's adaptive behavior skills. Notably, the methylation states in the institutionalized children were opposite to those that seemed to be advantageous for adaptive behavior and the development of everyday skills that, in turn, may indicate that specific epigenetic states may underlie behavioral differences, namely a lower adaptive level observed in children residing in orphanages.

Four significant associations were found for the communication and motor skills domains; for two of these, the tentative causal mechanism can be hypothesized. First, the VABS Motor Skills domain showed a significant negative association with the methylation level of a CpG related to promoter of the *LTBP1* gene, which controls and directs the activity of transforming growth factor beta (TGFB). TGFB plays an important role in the sustained production of collagen and in bone remodeling that, in turn, might be crucial for motor skills, which are partly related to musculoskeletal system development. Second, an association between lower methylation levels of an olfactory receptor *OR4K17* (that might be related to increased expression) and higher scores on the communication domain was found; olfactory function is a significant component of establishing interpersonal relationships and social network [90], especially in the context of early development [91].

### Cumulative effect of the institutionalization on children's adaptive behavior and epigenetic states

Despite the identified significant differences in behavioral and epigenetic profiles between children raised in institutions and children raised in biological families, we would like to exert special care in attributing these differences to the effects of institutional care. As we have pointed out, we cannot exclude that other factors may contribute to these differences, such as prenatal conditions that have not been reported in children's medical histories, or an experience of parental neglect that some children may experience before they have been removed from their families and placed into institutional care, among others. To strengthen the argument that institutionalization may have a causal relationship with different facets of child development, we explore the potential cumulative effect of institutionalization on children's adaptive behavior and epigenetic states. To this end, we performed two sets of association analyses, in which the duration of exposure (institutionalization duration in months) controlling for child gender and age at entry into institutional care was considered as a predictor of children's adaptive behavior skills and epigenetic status.

First, we conducted an association analysis at the level of individual variability of adaptive behavior scores and methylation measurements of 164 CpG sites that we defined as differentially methylated CpGs in the institutionalized children. Possibly due to small effect sizes and limited sample size, we did not find statistically significant associations between the duration of institutionalization and children's adaptive behaviors; yet, all VABS domain scores were consistently negatively associated with exposure duration. In addition, at least three methylation markers had a nominally significant association with the duration of institutionalization: an increase in the methylation of the *CNTNAP1—*a gene required for the organization of myelinated axons during brain development, and a decrease in the promoter methylation of a mitochondrial acetyltransferase gene *ACAT1* and the transcription factor *RB1* that might be attributed to the stress response and immune response pathways. Notably, previous studies found that institutionalization duration was associated with the downmethylation of stress-related genes, specifically *FKBP5* and *SLC6A4* genes in saliva cells [46].

Second, we explored the association of exposure duration with a composite index of the distance of institutionalized children's behavioral and epigenetic profiles from those of their family-reared peers. A significant positive association between the time a child had spent in an orphanage and the divergence of his behavior and epigenetic profiles from the “normative” profile was found. Thus, we showed that exposure duration seems to have a direct impact on both adaptive level and DNA methylation states in children residing in orphanages, making them more divergent from the relevant profiles of children raised by their biological families over time. Moreover, we observed that the deviations of behavioral and epigenetic profiles of institution-reared children from the normative profiles in the BFC group were positively related to each other. The determination coefficients estimated for the entire institutionalized cohort and for the subcohort of children raised in institutional care from birth indicated that in the IC group, up to 7–14% of the variance in the deviation of the adaptive behavior profile from the normative one can be explained by the deviation of their DNA methylation profile, thereby linking these indexes.

### Conclusions

We would like to emphasize that the effects of institutionalization might be overestimated, when the poor health and subnormal developmental trajectories of institution-reared children tend to be associated only with various facets of deprivation experiences in orphanages, without taking into consideration their inborn challenges. Because most children living in institutional care have lower, compared to typical children, indicators at entry (e.g., elevated frequencies of medical and social risk factors) that can shape their negative health trajectories (e.g., elevated frequencies of diseases and developmental delays). Nevertheless, focusing on the cohort of typically developing institution-reared infants, we found that, while developing within age norms, they showed statistically significantly lower adaptive behavior skills in multiple domains, and demonstrated a host of significant differences in their DNA epigenome-wide methylation profiles, both of which may be partly attributed to the characteristics of institutional environments.

Our study has two main limitations. First, the sample size of our cross-sectional EWAS was relatively small due to both difficulties with access to the population of children residing in state-run institutions and the specificity of the target cohort characterized by the elevated prevalence of health problems and developmental disorders that, given the stringent exclusion criteria in this study, significantly influenced participants' enrollment. Because of insufficiency of statistical power, many, interesting from our point of view, findings in the study were reported here as observed associations and tendencies. Second, a lack of relevant literature data on epigenomic profiling in cohorts of institutionalized children of similar age did not allow carrying out a comprehensive comparison to validate the findings and observations made in this study. Nevertheless, we found multiple parallels between our findings and those reported in research exploring the long-lasting epigenetic effects of early adverse environments. We believe that future research with additional cohorts of extended sample size will provide evidence supporting our findings and conclusions.

Despite the aforementioned limitations, to the best of our knowledge, this study is one of the first attempts to explore the impact of institutionalization on the epigenome and adaptive behavior and their interplay in young children at a critical stage of child development—infancy and toddlerhood. We believe that our study adds to the research documenting possible causal relationship between the epigenetic profile and its dynamics and various developmental outcomes in the context of individuals' health, behavior, and cognition. Altogether, the results of our work provide new data to the growing body of literature focused on the effects of early adversity, specifically institutionalization as a natural model of early psychosocial deprivation in humans, on child development and on the role of epigenetic regulation as a mechanism underlying these effects.

## Supporting information

S1 Fig Distributions of standard scores and Z-scores on four adaptive domains from the VABS assessment (Communication, Daily Living Skills, Socialization and Motor Skills) in the combined sample of n = 58 children (top and lateral panels), and the scatterplots for the associations between the standard and Z-scores. (TIF)

S2 Fig Heatmap illustrating the clustering of IC and BFC children based on blood cell-type composition estimates derived from DNA methylation data. (TIF)

S3 Fig Relationships between the two types of methylation values (beta-values and z-values). Top left corner represents the distribution of the pairwise Pearson's r coefficients for k = 164 epigenome-wide significant probes. As an example, we also present scatterplots for three individual CpGs that showed the maximum r close to 1 (the SMO gene), and for two CpGs that showed minimal r or whose beta-values have undergone the greatest transformation due to the correction on demographic variables (the SAR1B and RBM46 genes).
(TIF)

S1 Table Core demographics for the study sample: Children raised in institutional care (IC) and children raised by their biological families (BFC). Child age (in months), duration of institutionalization (in months) at the time of blood draw (Age_EPI_) and at the time of assessment (Age_VABS_) are presented.
(XLSX)

S2 Table Individual scores for the four VABS adaptive behavior domains for institutionalized children (IC; N = 29) and children raised in biological families (BFC; N = 25). SS–standard score.
(XLSX)

S3 Table Blood cell type distributions in IC and BFC children (individual data). (XLSX)

S4 Table CpG sites differentially methylated in IC group of children, compared to the BFC group. Individual methylation levels are represented in original beta-values and in betas adjusted on demographic variables, z-values. CpG sites annotation corresponds to Illumina's manifest for the MethylationEPIC microarray. Inter-group differences in CpG methylation levels are presented in fold change units.
(XLSX)

S5 Table List of 172 genes with known functions, which were significantly differentially methylated in the IC group; 87 genes that had a significant (STRING confidence score > .400) interaction with at least one other gene from the list are shown in bold. (XLSX)

S6 Table Illumina MethylationEPIC probes localized to the CpG island associated with promoter of the glucocorticoid receptor gene, NR3C1, which showed a significant difference (nominal p-value < .05) in methylation levels between the IC and the BFC group. (XLSX)

S7 Table List of 78 DMESs, whose methylation levels (z-values) had a significant (nominal P-value < .05) correlation with Z-scores for at least one of the four examined adaptive behavior domains according to VABS. Spearman rank correlation coefficients are presented. Coefficients with p < .05 are shown in red; coefficients with p < .01 are shown in bold; the coefficients that survived corrections for multiple testing are shown in grey.
(XLSX)
