# Supplementary figures and images for: Effects of early social deprivation on epigenetic statuses and adaptive behavior of young children: A study based on a cohort of institutionalized infants and toddlers
Source: PLoS One. 2019 Mar 26;14(3):e0214285. doi: 10.1371/journal.pone.0214285 (PMC6435191; doi:10.1371/journal.pone.0214285)

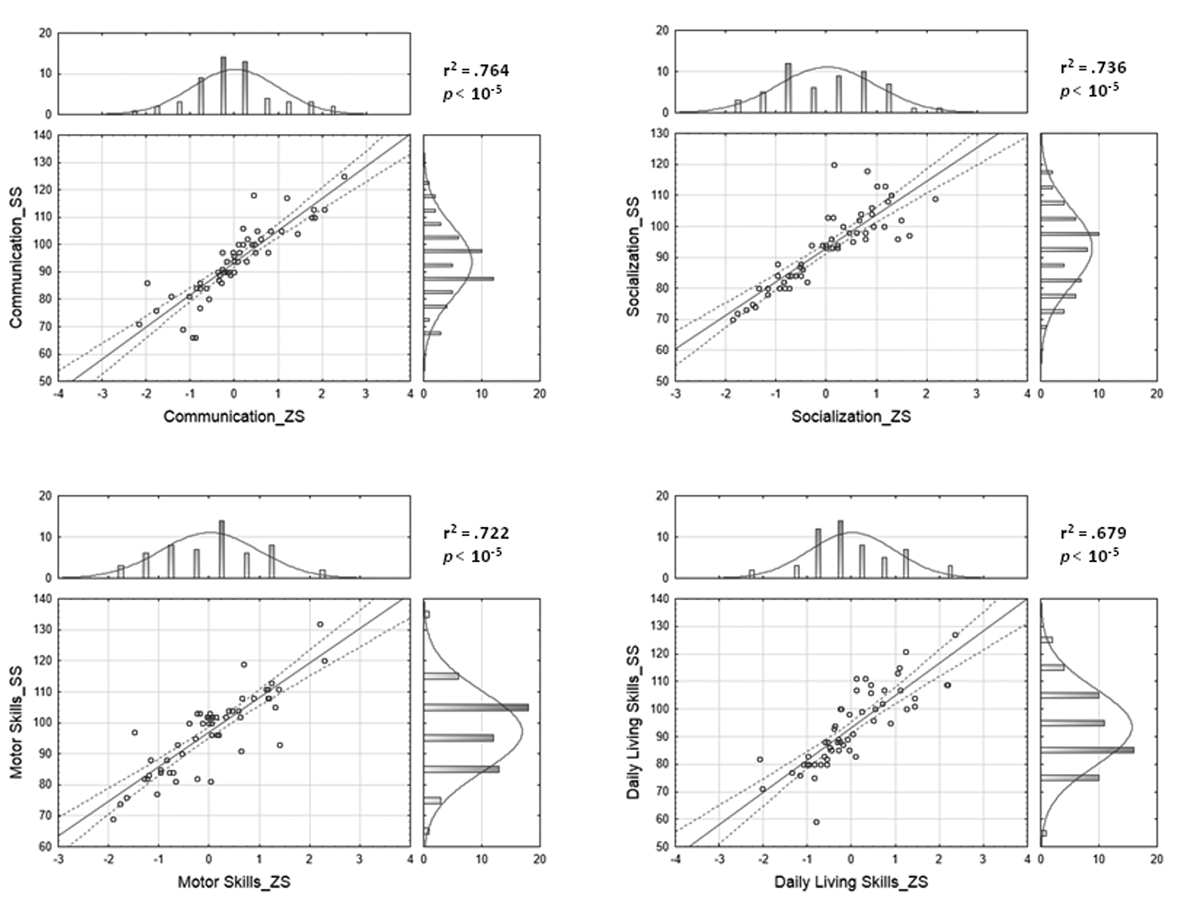

Supplement: S1 Fig — (TIF) [file pone.0214285.s001.tif]

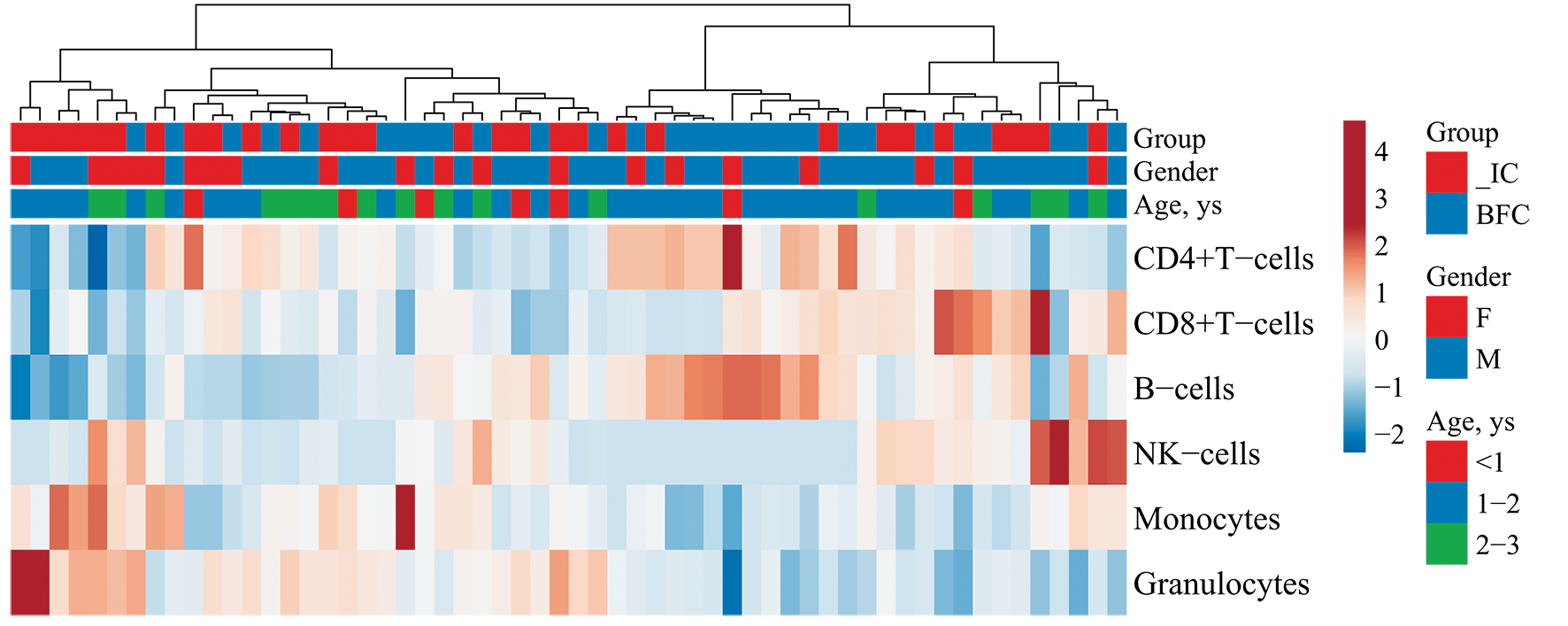

Supplement: S2 Fig — (TIF) [file pone.0214285.s002.tif]

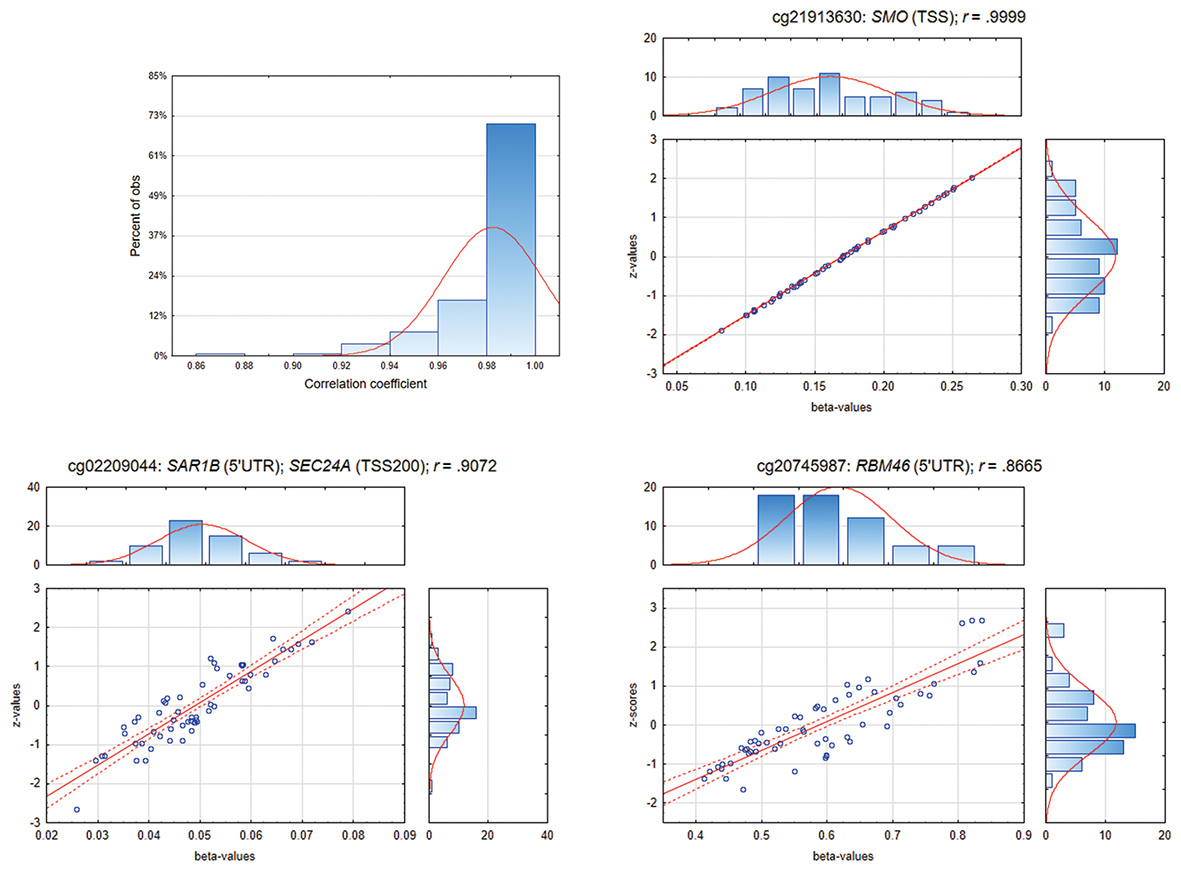

Supplement: S3 Fig — Top left corner represents the distribution of the pairwise Pearson's r coefficients for k = 164 epigenome-wide significant probes. As an example, we also present scatterplots for three individual CpGs that showed the maximum r close to 1 (the SMO gene), and for two CpGs that showed minimal r or whose beta-values have undergone the greatest transformation due to the correction on demographic variables (the SAR1B and RBM46 genes). (TIF) [file pone.0214285.s003.tif]
